# Participation of the Cell Polarity Protein PALS1 to T-Cell Receptor-Mediated NF-κB Activation

**DOI:** 10.1371/journal.pone.0018159

**Published:** 2011-03-30

**Authors:** Gabrielle Carvalho, Konstantinos Poalas, Catherine Demian, Emeline Hatchi, Aimé Vazquez, Nicolas Bidère

**Affiliations:** 1 INSERM, UMR_S 1014, Hôpital Paul Brousse, Villejuif 94800, France; 2 Université Paris-Sud P11, Orsay 91400, France; University Paris Sud, France

## Abstract

**Background:**

Beside their established function in shaping cell architecture, some cell polarity proteins were proposed to participate to lymphocyte migration, homing, scanning, as well as activation following antigen receptor stimulation. Although PALS1 is a central component of the cell polarity network, its expression and function in lymphocytes remains unknown. Here we investigated whether PALS1 is present in T cells and whether it contributes to T Cell-Receptor (TCR)-mediated activation.

**Methodology/Principal Findings:**

By combining RT-PCR and immunoblot assays, we found that PALS1 is constitutively expressed in human T lymphocytes as well as in Jurkat T cells. siRNA-based knockdown of PALS1 hampered TCR-induced activation and optimal proliferation of lymphocyte. We further provide evidence that PALS1 depletion selectively hindered TCR-driven activation of the transcription factor NF-κB.

**Conclusions:**

The cell polarity protein PALS1 is expressed in T lymphocytes and participates to the optimal activation of NF-κB following TCR stimulation.

## Introduction

Establishment and maintenance of cell polarity is chiefly orchestrated by a tightly regulated interplay between three multi-protein complexes: i) Scribble (SCRIB)/Discs Large (Dlgh1)/Lethal giant larvae (Lgl) complex, ii) partitioning-defective (PAR) 3 and PAR6/ atypical protein kinase C (aPKC) complex, and iii) Crumbs (CRB)/ Protein Associated with Lin Seven 1 (PALS1)/ PALS1-associated tight junction protein (PATJ) complex [1], [2]. However, each complex is not exclusive, as PAR6 links PALS1 to PAR3/PAR6/aPKC [3]. In T lymphocytes, cell polarity proteins were shown to partition the leading edge from the uropod at the cell rear, and therefore participate to cell migration, homing, and scanning [4], [5], [6]. In addition, SCRIB and Dlgh1 are transiently recruited to the nascent immunological synapse formed with an antigen-presenting-cell (APC) [4]. Their depletion in lymphocytes has been associated with an alteration of antigen receptor-mediated activation [7], [8], [9], [10].

The adaptor PALS1 is crucial for cellular architecture as it maintains the apico-basal polarity in epithelial cells and authorizes indirect interactions between CRB and PATJ [11], [12]. Interestingly, Dlgh1 and PALS1 share a COOH-terminal part composed of a PSD-95/Dlg/ZO-1 (PDZ) domain followed by an SH3 domain adjacent to an inactive Guanylate kinase (GK) homology region [2]. This unique sequence of PDZ/SH3/GK defines the so-called membrane-associated guanylate kinase (MAGUK) proteins family, a group of molecules that serve as scaffolds to organize multi-protein signalosomes through their protein-protein interaction domains [13]. For example, the MAGUK-containing CARMA1 emerges as a central regulator of lymphocytes activation and proliferation downstream of antigen receptor stimulation [14]. Indeed, CARMA1 operates as scaffold to recruit the heterodimer BCL10/MALT1 (CBM complex), a key step for conveying NF-κB signaling [14], [15], [16]. In addition to its established role in polarity, Dlgh1 was shown to modulate lymphocyte proliferation upon T-cell receptor ligation, possibly through p38 recruitment or via the transcription factor NF-AT [9], [10], [17], [18].

Although the MAGUK PALS1 plays a central role in the establishment of cell polarity, its contribution to lymphocyte activation remains elusive [8]. Here we show that PALS1 mRNA and protein is expressed in human lymphocytes. Furthermore, knocking down of PALS1 with small interfering RNAs (siRNAs) led to a decreased proliferation of human T lymphocytes, resulting from a reduced activation of the transcription factor NF-κB.

## Results and Discussion

### PALS1 expression in T lymphocytes

**Figure 1:**
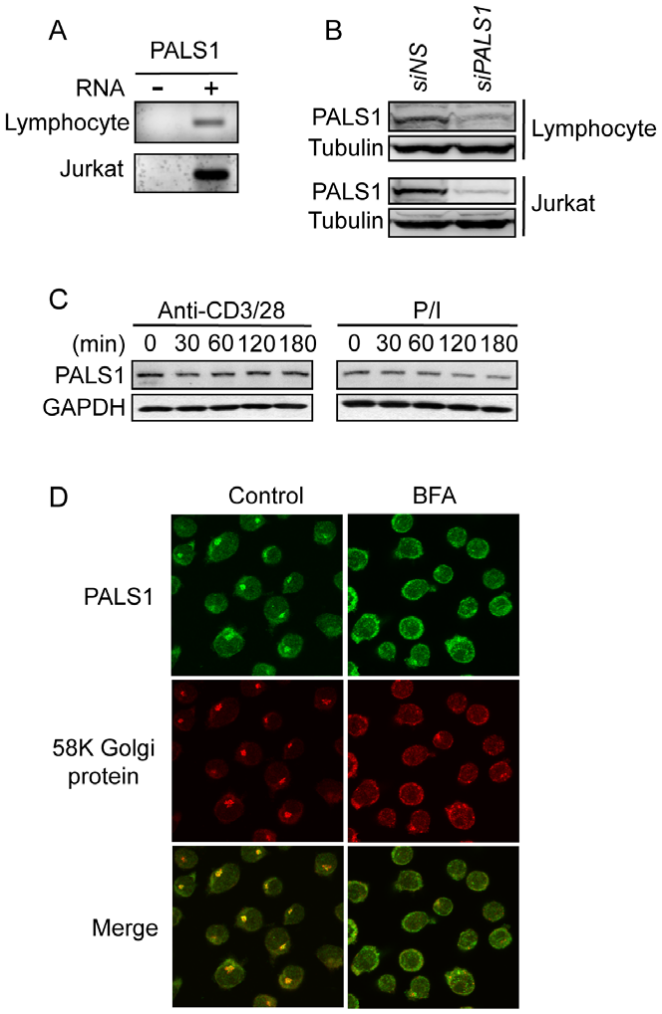
Expression of the cell polarity protein PALS1 in human T lymphocytes. **A,** Analysis of PALS1 mRNA in primary human T lymphocytes, and Jurkat cell line by Reverse Transcriptase Polymerase Chain Reaction (RT-PCR). **B,** Immunoblot analysis of PALS1 in cell lysates from human T lymphocytes and from Jurkat cells. The specificity of PALS1 antibodies was validated with lysates from cells transfected with siRNA against PALS1. Tubulin was used as a loading control. **C,** Immunoblot analysis of cell extracts from Jurkat cells stimulated as indicated with 1 µg.ml^−1^ anti-CD3 and anti-CD28, or with 40 ng.ml^−1^ PMA together with 300 ng.ml^−1^ ionomycin (P/I). GAPDH served as a loading control. **D,** Confocal microscopy pictures of PALS1 and 58K Golgi protein in Jurkat T lymphocytes either untreated or incubated with 10 µg.ml^−1^ Brefeldin A (BFA).

Although several cell polarity proteins have been characterized in lymphocytes [4], [5], PALS1 expression in T cells remains to be determined [8]. To address this question, we first performed RT-PCR analysis on resting human CD3^+^ T cells and Jurkat lymphocytes extracts, and detected mRNA for PALS1 (Figure 1A). These mRNA were efficiently translated into protein, as antibodies against PALS1 detected a band, which was absent from PALS1-siRNA transfected primary T lymphocytes lysates (Figure 1B). Similar results were obtained with Jurkat T cells (Figure 1B). Of note, PALS1 levels remained unchanged in cells stimulated with antibodies to CD3 and CD28, or with PMA and ionomycin (Figure 1C). We next investigated PALS1 subcellular location by confocal microscopy. In contrast to epithelial cells where it accumulate to tight junctions [12], PALS1 did not reach membrane domains and remains essentially cytosolic with punctuate structures. Additional staining revealed that these structures coalesced with the Golgi apparatus (Figure 1D and Figure S1). Accordingly, Brefeldin A-triggered disassembly of the Golgi apparatus also disrupted PALS1 punctuate structures (Figure 1D). This is reminiscent of PALS1 relocation to the Golgi apparatus in cells infected with SARS coronovirus [19]. Last, we observed that TCR-mediated stimulation only promoted a discrete redistribution of PALS1 within the cytosol of Jurkat cells (Figure S1). Altogether, our results suggest that similarly to Dlgh1, SCRIB, CRB3, and PKCζ [4], [5], the cell polarity protein PALS1 is expressed in lymphocytes at both mRNA and protein level.

### Requirement of PALS1 for optimal T cell activation and proliferation

**Figure 2:**
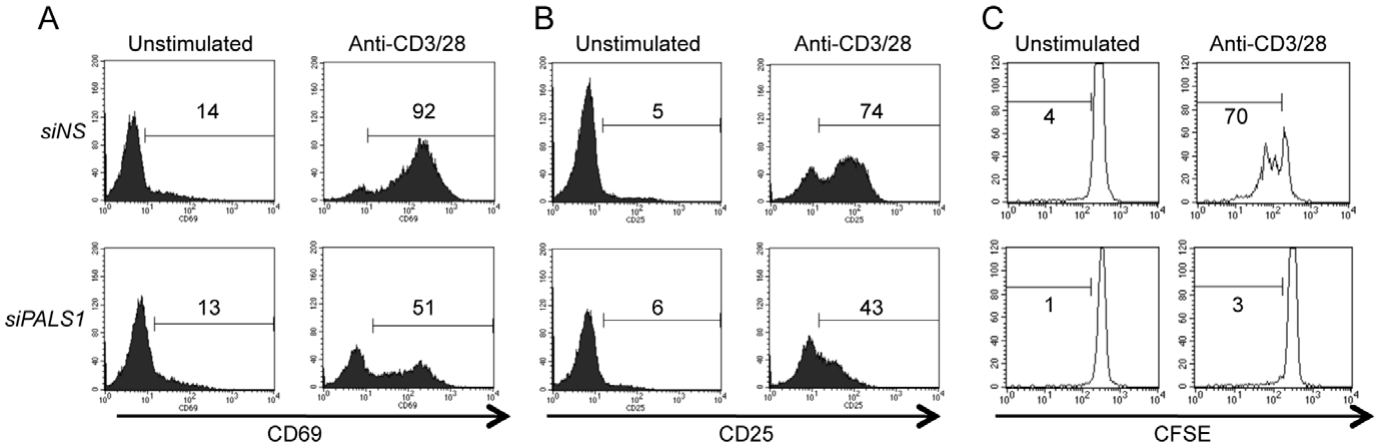
PALS1 requirement for optimal activation and proliferation in lymphocytes. **A and B,** Human peripheral blood T lymphocytes were transfected with siRNA for *PALS1* or nonspecific (*NS*) siRNA. Three days later, cells were stimulated with 1 µg.ml^−1^ anti-CD3 and anti-CD28. CD69 and CD25 induction at the cell surface were examined 6 hours and 16 hours post-stimulation, respectively. **C,** Cells as in (A) were loaded with Carboxyfluorescein Succinimidyl Ester (CFSE), and stimulated with 1 µg.ml^−1^ anti-CD3 and anti-CD28 for 72 hours. The percentage of CD69- and CD25-positive cells, and of dividing cells, is shown. These data are representative of four independent experiments.

Because SCRIB and Dlgh1 were proposed to modulate lymphocyte proliferation [7], [8], [9], [10], we evaluated whether PALS1 might also participate to T cell activation. To this end, peripheral blood lymphocytes (PBL) were purified on Ficoll-isopaque gradients. Primary human T cells were nucleofected for three days with siRNA targeting PALS1, prior stimulation with anti-CD3 and anti-CD28 antibodies. PALS1 knockdown led to a significant decrease in TCR-mediated induction of the activation markers CD69 and CD25 on cell surface (Figure 2A, B). This was accompanied by a reduction in Carboxyfluorescein Succinimydyl Ester (CFSE) dilution, which reflects cell proliferation (Figure 2C). Collectively, these data suggest that PALS1 participates to the optimal lymphocyte activation and subsequent proliferation upon TCR stimulation.

### Role of PALS1 in TCR-mediated signaling

**Figure 3:**
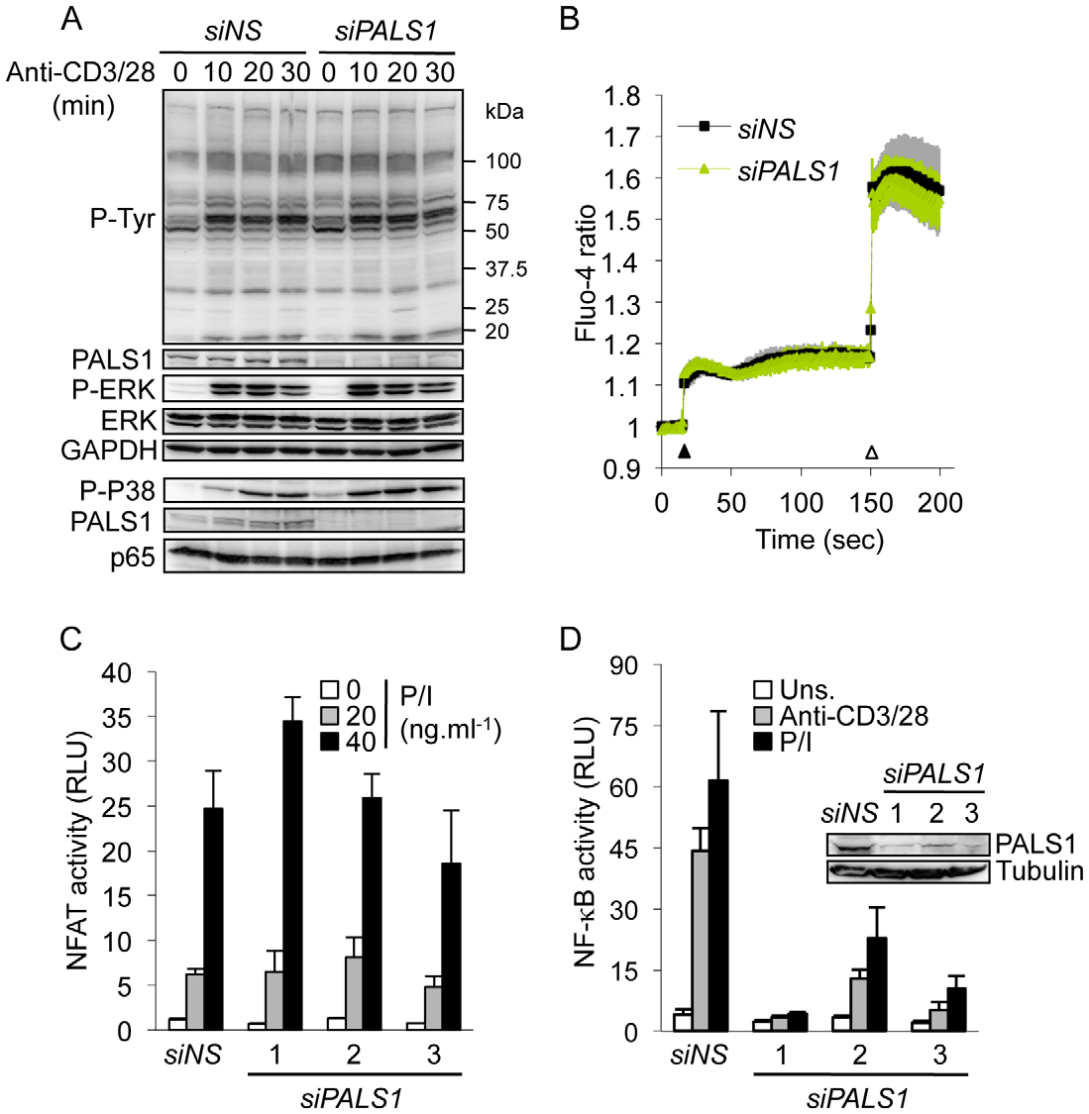
Role of PALS1 on early TCR-mediated signaling. **A,** Jurkat cells were transfected with nonspecific (*NS)*- and *PALS1*-siRNA, and left three days prior stimulation with 1 µg.ml^−1^ anti-CD3 and anti-CD28 for 0, 10, 20, and 30 min. Cell lysates were prepared and immunoblots were performed as indicated. GAPDH, and p65 served as loading controls. Five other experiments provided same results. **B,** *NS*- and *PALS1*-siRNA transfected Jurkat cells were loaded with the calcium-sensitive dye Fluo-4 and stimulated with 1 µg.ml^−1^ anti-CD3 (closed symbol), or with 1 µg.ml^−1^ ionomycin (open symbol). Shown is the mean ± s.d. of triplicate measurements (one out of two independent experiments). **C, D,** Jurkat lymphocytes were transfected with *NS*- or with three individual siRNA sequences targeting PALS1. After three days, cells were co-transfected with siRNA and with NF-AT or NF-κB firefly luciferase reporter gene together with a control Renilla plasmid for an additional 24 hours. Cells were then stimulated with 20 or 40 ng.ml^-1^ PMA and 300 ng.ml^−1^ ionomycin (P/I), or 0.5 µg.ml^−1^ anti-CD3 and anti-CD28. Histograms represent the mean ± s.d. of triplicate experiments. RLU, relative light units. The inset immunoblot shows the level of PALS1 knockdown. Data shown are representative of at least five independent experiments.

To further explore how PALS1 impacts on lymphocyte proliferation, early signaling pathways emanating from the TCR were examined in Jurkat cells transfected with PALS1 siRNA. We did not detect major alteration in the general pattern of tyrosine phosphorylation, or mitogen-activated protein kinase (MAPK) extracellular signal-regulated kinases (ERK) 1/2 phosphorylation upon TCR stimulation (Figure 3A). Only a slight but consistent increase in TCR-mediated phosphorylation of p38 was noted (Figure 3A). Moreover, CD3-induced calcium mobilization was largely normal in PALS1-knockdown Jurkat cells (Figure 3B).

We next analyzed TCR-mediated activation of NF-AT and NF-κB transcription factors. siRNA-treated Jurkat T cells were co-transfected with firefly luciferase constructs driven by NF-AT or NF-κB binding sequences and with a renilla luciferase control. PALS1 knockdown had only a marginal effect on NF-AT activity following stimulation with PMA and ionomycin, or with antibodies to CD3 and CD28 (Figure 3C and Figure S2). In sharp contrast, NF-κB activity was significantly reduced without PALS1 (Figure 3D). Interestingly, tumor necrosis factor-α (TNFα)-induced NF-κB activation remained essentially unaffected, underscoring the selective involvement of PALS1 in the TCR-NF-κB pathway (Figure S3). Altogether, our data unveiled an unexpected role for PALS1 in TCR-mediated NF-κB activation.

### PALS1 participates to the optimal activation of NF-κB upon TCR stimulation

**Figure 4:**
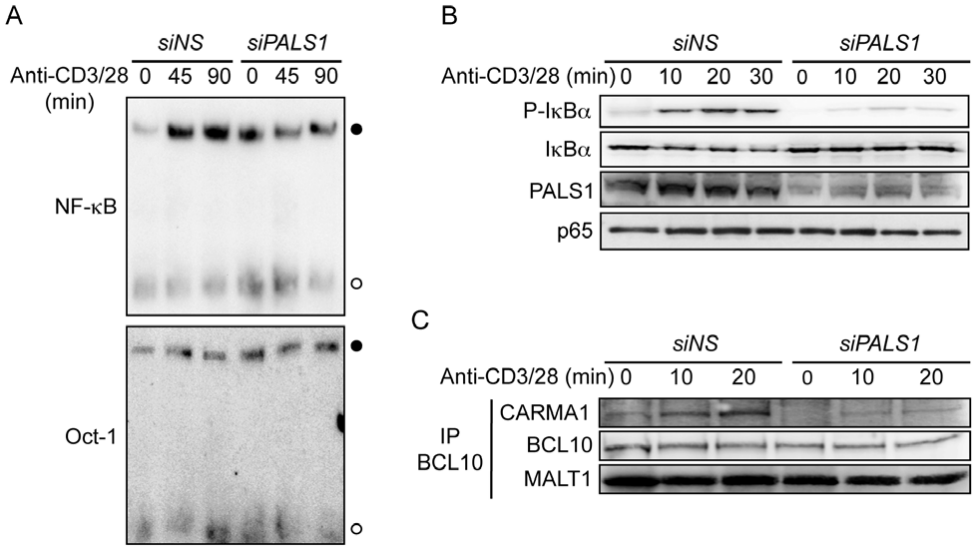
Involvement of PALS1 in TCR-mediated activation of NF-κB. **A,** Jurkat cells were transfected with nonspecific (*NS)*- and *PALS1*-siRNA. Three days later, cells were stimulated with 1 µg.ml^−1^ anti-CD3 and anti-CD28 for 0, 45, and 90 min. Nuclear extracts were prepared to analyze the binding of NF-κB and Oct-1 by electrophoretic mobility shift assays (EMSA) with specific probes (closed circles). Free probe is also indicated (open circles). **B,** Cells as in (A) were stimulated with 1 µg.ml^−1^ anti-CD3 and anti-CD28 for 0, 10, 20, and 30 min. Cell extracts were prepared and immunoblots were performed as indicated. **C,** Cells as in (A) were stimulated with 1 µg.ml^−1^ anti-CD3 and anti-CD28 for 0, 10, 20 min. BCL10 was immunoprecipitated (IP) from cell lysates, and the binding of CARMA1, and MALT1 was assessed by immunoblot. Data shown are representative of at least three independent experiments.

To gain insights on how PALS1 modulate NF-κB, we first investigated the transcription factor binding ability by electrophoretic mobility shift assay (EMSA). Less NF-κB bound to its specific probe in nuclei extracts from PALS1–siRNA transfected cells following TCR stimulation (Figure 4A). As expected, Oct-1 binding remained unchanged without PALS1. Consistent with a diminished NF-κB activity, both the phosphorylation and subsequent proteasomal degradation of NF-κB inhibitor, IkBα, were severely decreased in the absence of PALS1 (Figure 4B). Because TCR-induced NF-κB activation relies on the assembly of the CBM complex [15], BCL10 was immunoprecipitated from nonspecific (*NS*-) and *PALS1*-siRNA transfected Jurkat cells. MALT1, which forms an heterodimer with BCL10, coprecipitated with BCL10 regardless of stimulation. Although PALS1 was not found bound to BCL10, its absence diminished CARMA1 recruitment (Figure 4C, and data not shown). Hence, our data suggest that PALS1 participates to the optimal translocation and activation of NF-κB upon TCR stimulation, possibly by favoring the CBM assembly.

### Role of PALS1-associated proteins during TCR-mediated NF-κB

**Figure 5:**
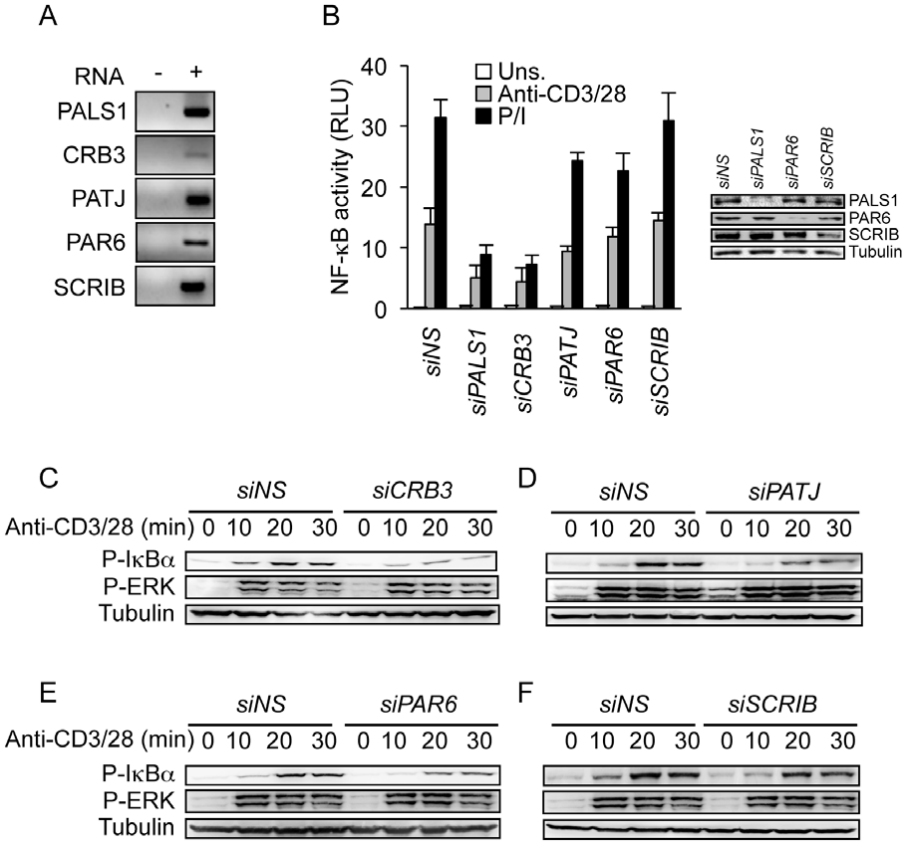
Role of PALS1 cell polarity partners in NF-κB signaling. **A,** Expression of cell polarity proteins PALS1, CRB3, PATJ, PAR6, and SCRIB by RT-PCR in Jurkat T lymphocytes. **B,** Jurkat were transfected with nonspecific (*NS*)-, *CRB3*-, *PAR6*-, *PATJ*- and *SCRIB*-siRNA. After three days, cells were then co-transfected with siRNA and with NF-κB firefly luciferase reporter gene together with a control Renilla plasmid. 24 hours later, cells were stimulated with 0.5 µg.ml^−1^ anti-CD3/CD28 or with 20 ng.ml^−1^ PMA and 300 ng.ml^−1^ ionomycin (P/I). Shown is the mean ± s.d. of triplicate experiments. RLU, relative light units. **C–F,** Immunoblots as indicated of *NS*-, *CRB3*-, *PAR6*-, *PATJ*- and *SCRIB*-siRNA transfected Jurkat cells stimulated with 1 µg.ml^−1^ anti-CD3 and anti -CD28 for 0, 10, 20, and 30 min. Data shown are representative of three independent experiments.

Since PALS1 nucleates a ternary complex containing CRB3 and PATJ, and further binds PAR6 to maintain cell polarity [3], [20], [21], their contribution to TCR-mediated NF-κB was evaluated. Similarly to PALS1, mRNA for PATJ, CRB3, PAR6, were efficiently detected by RT-PCR (Figure 5A). The same hold true for the unrelated cell polarity protein SCRIB (Figure 5A). siRNA-based knockdown of PALS1 and CRB3 significantly decreased NF-κB activation in cells treated by antibodies against CD3 and CD28, or with a mixture of PMA and ionomycin. Although less dramatic, similar results were observed with PAR6 or PATJ knockdown. By contrast, NF-κB was normally activated in the absence of SCRIB (Figure 5B). In agreement, IkBα phosphorylation was diminished in lysates from CRB3-depleted cells, and to a lesser extent from PATJ- or PAR6-siRNA transfected cells, and not from SCRIB-depleted cells. Again, ERK phosphorylation occurred normally (Figure 5C, D, E, and F). Altogether, our data suggest that PALS1 implication in the TCR-NF-κB pathway is inextricably linked to its cell polarity partners.

In summary, our data show that the cell polarity protein PALS1 is expressed in lymphocytes and contributes to their optimal activation. Although Dlgh1 and SCRIB were proposed to modulate NF-AT or p38 [9], [10], [17], [18] and NF-AT [7] respectively, a distinct scenario likely occurs for PALS1. Our results support a model in which PALS1 participates to NF-κB activation, upstream of IkBα phosphorylation and degradation. However, how precisely PALS1 modulates NF-κB remains unclear. Because MAGUK function as scaffold units to organize and integrate multi-molecular signaling complexes [13], it is tempting to speculate that PALS1 nucleates its own signalosome. For example, CARMA1 anchors a >900 kDa complex including the heterodimer BCL10/MALT1 [22], and Dlgh1 was reported to bind to Lck, Zap70, Wasp [17], and p38 [10]. In our hands, PALS1 did not integrate the CBM, but its absence reduced CARMA1 binding to BCL10. It will therefore be interesting to identify PALS1 partners in the context of lymphocyte activation. In line with this, CRB3, PATJ and PAR6, which all bound PALS1 to maintain cell polarity [2], also participate to NF-κB signaling upon TCR ligation in lymphocytes, and might therefore complex with PALS1 in lymphocytes. Altogether, our results strengthen the unexpected function of cell polarity proteins in lymphocyte proliferation [7], [8], [9], [10], and unveil an original role for PALS1 during TCR-mediated NF-κB activation.

## Materials and Methods

### Cell culture and reagents

Jurkat T cells E6.1 were purchased from ATCC. CD3^+^ human T lymphocytes from healthy donors (Etablissement Francais du Sang) were isolated with the MidiMacs system (Miltenyi Biotec). Cells were activated with a mixture of soluble anti-CD3ε (HIT3a, BD Biosciences) and anti-CD28 (BD Biosciences), or with 20–40 ng.ml^−1^ phorbol 12-myristate 13-acetate (PMA, Sigma) and 300 ng.ml^−1^ ionomycin (Calbiochem). Carboxyfluorescein Succinimydyl Ester (CFSE) and Brefeldin A were purchased from Sigma, and the calcium-sensitive dye Fluo-4 was from Invitrogen.

### Cell lysates preparation, immunoprecipitations, and immunoblots

Cells were washed twice with PBS 1X and lysed with 50 mM Tris pH 7.4, 150 mM NaCl, 1% Triton X-100, 1% Igepal, 2 mM EDTA, supplemented with complete protease inhibitors (Roche). Lysates were cleared by a centrifugation at 10,000*g* at 4^o^C, and protein concentration determined (micro BCA kit, Pierce). Samples were resolved on 5–20% SDS-PAGE gels and transferred to nitrocellulose membranes (Amersham). For Immunoprecipitations, samples were precleared with protein G-sepharose beads (Roche) for 30 min prior to overnight incubation with antibodies and additional protein G-sepharose beads at 4°C, as previously described [23]. Antibodies to BCL10 (A-6), IkBα (C-21), MALT1 (B-12), Tubulin (TU-02), PALS1 (H-250), SCRIB (C-6), PAR6 (G-9) and p65 (C-20) were purchased from Santa Cruz. Phospho-specific antibodies against IkBα, ERK, p38, and antibodies to CARMA1 and to ERK were from Cell Signaling Technologies. Anti-phosphorylated Tyrosine (4G10, Millipore), anti-GAPDH (Sigma), and Immobilon (Millipore) chemiluminescent substrates were also used.

### Luciferase assays

Firefly luciferase constructs downstream of promoters for NF-κB or NF-AT were co-transfected with renilla luciferase pRL-TK (Int^-^) plasmid (Promega). Luciferase activities were analyzed using the Dual-Luciferase Kit (Promega), with firefly fluorescence units normalized to renilla luciferase fluorescence units (BMG microplate reader).

### siRNA and transfections

All siRNA used were from Invitrogen (Stealth). PALS1.1, 5′-CCAAGGAAACAGUAAUCCAUGUAAA-3′; PALS1.2, 5′-GAGGAGAUCUUAACCUAUGAGGAAA-3′; PALS1.3, 5′-CAGAACAAUGGCCACUACUUUGAUA-3′; CRB3, 5′-CCAUCACUGCUAUCAUCGUGGUCUU-3′; PATJ, 5′-GCAUGAAUUUCUGACUCCUAGAUUG-3′; SCRIB, 5′-UGGGAGGCAACGAUCUGGAAGUGCU-3′; PAR6 5′-GAGCGGGUUCCAGGAAUCUUCAUCU-3′. Jurkat cells were transfected by electroporation with a BTX ECM 830 apparatus (BTX, Harvard Apparatus), as previously described [16]. For primary cells, PBL were purified from blood on Ficoll-isopaque gradients. PBL were nucleofected with the Nucleofactor system and T cell solution (Amaxa, program U14), and left for three days in culture medium prior treatment.

### Nuclear protein extraction and electrophoretic mobility shift assay (EMSA)

4 µg of nuclear extracts from Jurkat cells were examined for NF-κB- and Oct1-binding activity by electromobility shift assay (Panomics kit). Samples were resolved on a 6% native polyacrylamide DNA retardation gel in 0.5X TBE buffer and analyzed using a FUJI LA4000 system.

### Reverse transcriptase PCR

2 µg of total RNA from purified human blood T cells or Jurkat were used for the RT-PCR reactions (RNeasy and OneStep kits, Quiagen). Primers were designed as follows: PALS1: Forward (F), 5′-CTCCTTCATGCAACAGACCA-3′ and Backward (B), 5′-CACTTTTACTGGCCCACGAT-3′; CRB3: F, 5′-CACCTGCTCCTCGCTACTG-3′ and B, 5′-CACTGTTTTGCCTTCATCCA-3′; PATJ: F, 5′-CAACGAGCATCCTGACTGAA-3′ and B, 5′-GGCGTGGTTGTGAGGACTAT-3′; PAR6: F, 5′-GTTGCCAACAGCCATAACCT-3′ and B, 5′-CAGGTCACTGCTGTCATCGT-3′; SCRIB: F, 5′-CGCAAGGACACACCTCACTA-3′ and B, 5′-CCTCCTCCTGAGGACTACCC-3′.

### Confocal microscopy

Cells were left for 10 min on poly-lysine coated slides (Thermo Scientific) prior fixation with PBS1X containing 4% paraformaldehyde. For TCR crosslinking experiments, cells were incubated with 5 µg.ml^−1^ anti-CD3 at 4°C for 15 min. After two washes, cells were incubated with 5 µg.ml^−1^ of goat anti-mouse (Jackson) for 20 min either at 4°C or 37°C. To disassemble Golgi apparatus, cells were treated with 10 µg.ml^−1^ Brefeldin A for 60 min. Samples were permeabilized with 0.05% Triton-X100 in PBS1X for 5 min, and non-specific sites blocked with 10% FCS in PBS1X. Antibodies used were: PALS1 (Millipore), 58K Golgi (Abcam), Alexa-488 conjugated goat anti-rabbit IgG or Alexa-594 conjugated goat anti-mouse IgG (Invitrogen). Samples were analyzed using a Leica confocal microscope SP6.

### Cell surface staining

Cells were incubated for 30 min at 4°C with FITC- and PE-conjugated antibodies against CD25 and CD69 (ImmunoTools) and the respective isotype controls in PBS containing 0.5% BSA. After one wash with ice-cold PBS-BSA, cells were analyzed by flow cytometry with a FACSCalibur (BD Biosciences).

## Supporting Information

Figure S1 **Impact of stimulation on PALS1 subcellular location.** **A**, Jurkat were stimulated 30 min with 40 ng.ml^−1^ PMA and 300 ng.ml^−1^ ionomycin. Shown are confocal microscopy pictures of PALS1 and 58K golgi protein. **B**, CD3 was crosslinked at the plasma membrane of Jurkat cells either at 4 or 37°C for 20 min. Micrographs show double staining for CD3 and PALS1.
(EPS)

Figure S2 **PALS1 is dispensable for TCR-mediated NF-AT activation.** Jurkat lymphocytes were transfected with NS- or with PALS1-siRNA. After three days, cells were co-transfected with siRNA and with NF-AT firefly luciferase reporter gene together with a control Renilla plasmid for an additional 24 hours. Cells were then stimulated with 20 ng.ml^−1^ PMA and 300 ng.ml^−1^ ionomycin (P/I), or with 1 µg.ml^−1^ anti-CD3 and anti-CD28. Histograms represent the mean ± s.d. of triplicate experiments. RLU, relative light units.
(EPS)

Figure S3 **Role of PALS1 cell polarity partners on TNFα-induced NF-κB activation.** **A**, Jurkat were transfected with nonspecific (NS)-, PALS1-, CRB3-, PAR6-, and PATJ-siRNA for three days. Cells were then co-transfected with siRNA and with NF-κB firefly luciferase reporter gene together with a control Renilla plasmid. 24 hours later, cells were stimulated with 10 ng.ml^−1^ TNFα for 6 hours. Shown is the mean ± s.d. of triplicate experiments. RLU, relative light units.
(EPS)

## References

[1] Iden S, Collard JG (2008). Crosstalk between small GTPases and polarity proteins in cell polarization.. Nat Rev Mol Cell Biol.

[2] Assemat E, Bazellieres E, Pallesi-Pocachard E, Le Bivic A, Massey-Harroche D (2008). Polarity complex proteins.. Biochim Biophys Acta.

[3] Hurd TW, Gao L, Roh MH, Macara IG, Margolis B (2003). Direct interaction of two polarity complexes implicated in epithelial tight junction assembly.. Nat Cell Biol.

[4] Ludford-Menting MJ, Oliaro J, Sacirbegovic F, Cheah ET, Pedersen N (2005). A network of PDZ-containing proteins regulates T cell polarity and morphology during migration and immunological synapse formation.. Immunity.

[5] Real E, Faure S, Donnadieu E, Delon J (2007). Cutting edge: Atypical PKCs regulate T lymphocyte polarity and scanning behavior.. J Immunol.

[6] Hawkins ED, Russell SM (2008). Upsides and downsides to polarity and asymmetric cell division in leukemia.. Oncogene.

[7] Arpin-Andre C, Mesnard JM (2007). The PDZ domain-binding motif of the human T cell leukemia virus type 1 tax protein induces mislocalization of the tumor suppressor hScrib in T cells.. J Biol Chem.

[8] Krummel MF, Macara I (2006). Maintenance and modulation of T cell polarity.. Nat Immunol.

[9] Stephenson LM, Sammut B, Graham DB, Chan-Wang J, Brim KL (2007). DLGH1 is a negative regulator of T-lymphocyte proliferation.. Mol Cell Biol.

[10] Round JL, Humphries LA, Tomassian T, Mittelstadt P, Zhang M (2007). Scaffold protein Dlgh1 coordinates alternative p38 kinase activation, directing T cell receptor signals toward NFAT but not NF-kappaB transcription factors.. Nat Immunol.

[11] Kamberov E, Makarova O, Roh M, Liu A, Karnak D (2000). Molecular cloning and characterization of Pals, proteins associated with mLin-7.. J Biol Chem.

[12] Roh MH, Makarova O, Liu CJ, Shin K, Lee S (2002). The Maguk protein, Pals1, functions as an adapter, linking mammalian homologues of Crumbs and Discs Lost.. J Cell Biol.

[13] Funke L, Dakoji S, Bredt DS (2005). Membrane-associated guanylate kinases regulate adhesion and plasticity at cell junctions.. Annu Rev Biochem.

[14] Blonska M, Lin X (2009). CARMA1-mediated NF-kappaB and JNK activation in lymphocytes.. Immunol Rev.

[15] Thome M, Charton JE, Pelzer C, Hailfinger S (2010). Antigen receptor signaling to NF-kappaB via CARMA1, BCL10, and MALT1.. Cold Spring Harb Perspect Biol.

[16] Carvalho G, Le Guelte A, Demian C, Vazquez A, Gavard J (2010). Interplay between BCL10, MALT1 and IkappaBalpha during T-cell-receptor-mediated NFkappaB activation.. J Cell Sci.

[17] Round JL, Tomassian T, Zhang M, Patel V, Schoenberger SP (2005). Dlgh1 coordinates actin polymerization, synaptic T cell receptor and lipid raft aggregation, and effector function in T cells.. J Exp Med.

[18] Xavier R, Rabizadeh S, Ishiguro K, Andre N, Ortiz JB (2004). Discs large (Dlg1) complexes in lymphocyte activation.. J Cell Biol.

[19] Teoh KT, Siu YL, Chan WL, Schluter MA, Liu CJ (2010). The SARS coronavirus E protein interacts with PALS1 and alters tight junction formation and epithelial morphogenesis.. Mol Biol Cell.

[20] Shin K, Straight S, Margolis B (2005). PATJ regulates tight junction formation and polarity in mammalian epithelial cells.. J Cell Biol.

[21] Roh MH, Fan S, Liu CJ, Margolis B (2003). The Crumbs3-Pals1 complex participates in the establishment of polarity in mammalian epithelial cells.. J Cell Sci.

[22] Oeckinghaus A, Wegener E, Welteke V, Ferch U, Arslan SC (2007). Malt1 ubiquitination triggers NF-kappaB signaling upon T-cell activation.. EMBO J.

[23] Bidere N, Ngo VN, Lee J, Collins C, Zheng L (2009). Casein kinase 1alpha governs antigen-receptor-induced NF-kappaB activation and human lymphoma cell survival.. Nature.

